# Association of Increased Cardio-Ankle Vascular Index (CAVI) with Echocardiographically Impaired Diastolic Dysfunction and Low Klotho Levels in Kidney Transplant Patients

**DOI:** 10.3390/jcm15072727

**Published:** 2026-04-03

**Authors:** Taliha Güçlü Kantar, Belda Dursun, Ahmet Baki Yağcı, İsmail Doğu Kılıç, İlyas Cihan Sevgican, Hande Şenol, Çağrı Ergin

**Affiliations:** 1 Department of Internal Medicine, School of Medicine, Pamukkale University, Denizli 20070, Türkiye; 2 Department of Nephrology, School of Medicine, Pamukkale University, Denizli 20070, Türkiye; belda.dursun@gmail.com; 3 Department of Radiology, School of Medicine, Pamukkale University, Denizli 20070, Türkiye; bakiyagci@yahoo.com; 4 Department of Cardiology, School of Medicine, Pamukkale University, Denizli 20070, Türkiye; idogukilic@yahoo.com (İ.D.K.); c7sevgican@gmail.com (İ.C.S.); 5 Department of Biostatistics, School of Medicine, Pamukkale University, Denizli 20070, Türkiye; handesenol@gmail.com; 6 Department of Microbiology, School of Medicine, Pamukkale University, Denizli 20070, Türkiye; cagri@pau.edu.tr

**Keywords:** kidney transplantation, CAVI, arterial stiffness, klotho

## Abstract

**Background and Objectives:** Cardiovascular disease is the leading cause of mortality after kidney transplantation. “Cardio-ankle vascular index” (CAVI), a recently devised technique whose utility for evaluation of cardiac risk in kidney transplantation is not well known. We investigated the associations of CAVI with echocardiographic assessment of cardiac functions and atherosclerotic parameters including FGF-23 and klotho. **Materials and Methods:** Two age- and gender-matched groups were subjects in the study. Group 1 included 75 KT patients with kidney transplant durations of at least 2 years; group 2 included 55 non-uremic controls. All participants underwent CAVI measurement and echocardiographic assessment. Atherosclerosis-associated parameters were determined using standard methods. **Results:** CAVI levels were similar between transplant patients and controls (7.26 ± 1.68 vs. 7.02 ± 1.3 m/sec); however, the percentage of subjects with pathological CAVI score (>8) was higher in transplant group (*p* = 0.077). Echocardiographic parameters displayed a significant increase in KT patients with higher CAVI scores. Low klotho levels were found to be significantly correlated to increased CAVI scores. Independent predictors of CAVI levels, as revealed by stepwise regression analysis, included high E/e’ (*p* = 0.012) and low klotho (*p* = 0.001). **Conclusions:** Our findings showed that higher CAVI scores still persist to some extent even after successful kidney transplantation and are independently linked to impaired E/e’ levels, which is an indicator of impaired diastolic dysfunction and low klotho levels. These findings underscore the importance of cardiovascular risk control in KT patients and suggest a possible role for klotho-mediated mechanisms in the development of arterial stiffness.

## 1. Introduction

Despite a 10–20-year increase in life expectancy following successful renal transplantation [1], atherosclerotic cardiovascular disease (CVD) significantly reduces the survival of those patients [2]. Among individuals experiencing chronic kidney disease, including renal transplant patients, the presence of heightened rates of established cardiovascular risk factors (hypertension, diabetes, hyperlipidemia) and non-traditional factors (inflammation, oxidative stress, malnutrition, vascular calcification) predisposes individuals to increased cardiovascular morbidity and mortality. The most important mechanism underlying cardiovascular diseases are processes including “left ventricular hypertrophy” (LVH), aortic stiffness and systolic–diastolic dysfunction due to hypertension and atherosclerosis, which are frequently seen in those patients. Risks of hypertension, cardiovascular events, and all-cause mortality are increased due to heightened arterial stiffness, resulting in augmented left ventricular (LV) wall thickness and compromised diastolic relaxation [3].

In the evaluation using derivatives of “pulse wave velocity” (PWV), previous studies have demonstrated varied correlations between arterial stiffness and the function and structure of the left ventricle (LV) [3,4]. The association with cardiovascular events is evident in the frequently used carotid-femoral PWV, but this measure does not encompass the elastic characteristics of the proximal aorta [4]. “Cardio-ankle vascular index” (CAVI) is a recent metric to predict possible cardiovascular disease risk as a novel non-invasive indicator of arterial stiffness. Conventional noninvasive arterial stiffness parameters such as PWV and baPWV may not provide reliable results in patients with kidney diseases due to increased blood pressure. Also, CAVI appears to be the preferred parameter due to its independence of changes in blood pressure [5]. With the correlation of arterial stiffness measurements evaluated by various non-invasive methods with CV disease findings such as LVH and “endothelial dysfunction” (ED) in renal transplant patients and the general population, end-stage renal disease (ESRD) has been shown in numerous studies. Currently, the literature does not contain any research that delves into the relationship between LV function and CAVI in renal transplant patients.

Vascular calcification, which is recognized by osteoblastic differentiation of vascular smooth muscle cells, is among the mechanisms leading to arterial stiffness in chronic kidney disease, and its pathogenesis is highly complex and includes traditional cardiovascular risk factors as well as mineral metabolism disorders (elevated calcium, phosphate, alkaline phosphatase), elevated parathormone (PTH), inflammation, oxidative stress and malnutrition [6]. Klotho was initially identified as an antiaging gene predominantly expressed in the kidney, parathyroid, and choroid plexus. Klotho plays a role in nitric oxide production in the endothelium, maintaining endothelial integrity and regulating its permeability [7]. There is an association between decreased klotho levels and atherosclerotic diseases [8]. Klotho is a “fibroblast growth factor-23” (FGF-23) cofactor that acts by increasing the receptor sensitivity of FGF-23. Overexpression of klotho has been found to reduce atherosclerosis by decreasing calcification in the aorta [9]. A decrease in klotho can lead to diseases associated with aging, such as hypertension [10], diabetes [11], chronic kidney disease (CKD) and cardiovascular disease [12]. The treatment considered most optimal for patients with ESRD is renal transplantation, which has been found in recent studies to influence klotho levels [13]. However, research on the role of klotho in renal transplantation is still insufficient.

It is still not clear whether the arterial stiffness mechanism regresses significantly after renal transplantation and the factors underlying its pathogenesis in that specific population are not well established. Therefore, evaluation of CAVI, a recently devised technique for measuring arterial stiffness, was conducted in both “stable kidney transplant” (KT) recipients and non-CKD controls, and its relation to echocardiographically identified heart function abnormalities and atherosclerosis-associated parameters, including FGF-23 and soluble klotho, were determined.

## 2. Materials and Methods

### 2.1. Patient Data

The study included 75 renal transplant patients with a history of at least two years of renal transplantation without known cardiovascular disease and a control group of 55 patients without renal dysfunction, diabetes and any cardiac disease history (coronary bypass, MI, angina, etc.). In our center, these patients were transplanted only between relatives up to the 4th degree and spouses in accordance with the organ transplantation laws in Turkey. In total, 72% (54 patients) were transplanted from cadaver and 28% (21 patients) from living donors. Stable patients who had undergone a renal transplantation process of at least two years were included. Induction therapy for KT patients involved the administration of IL-2 receptor antagonist and intravenous methylprednisolone followed by oral prednisolone, calcineurin inhibitors (“tacrolimus or cyclosporin A”) and mycophenolate mofetil as a standard immunosuppressive treatment regimen. Individuals in the control group were chosen to match the criteria of age and gender. Those with documented atherosclerotic cardiovascular disease, peripheral vascular disease, and those with symptoms of these diseases were not included in the study. It was recorded whether they had a smoking habit or not. Patients with suspected acute kidney insufficiency (AKI), history of contrast agent exposure (within the last month), malignancy, infectious symptoms and hemodynamically unstable patients were excluded.

### 2.2. Laboratory Analyzes

Blood and urine samples of all subjects included in the study were taken at 8 am after a 12 h fasting. For FGF-23 and klotho, blood collected in anticoagulant-free tubes was spun at 5000 rpm for 5 min and serum was separated and stored at −80 degrees Celsius to be studied together. All samples were analyzed by ELISA according to the manufacturers’ protocols and the instructions specified in the corresponding kit inserts. For FGF-23, the “Human FGF-23 (C-Term) ELISA Kit” (cat no.201-12-8847; SunredBio, Shanghai, China) kit was used; for klotho, the “Human Soluble Alpha-Klotho ELISA Kit” (cat no.201-12-5446; SunredBio, Shanghai, China) kit was used. Other laboratory analyses were performed using standard methods.

### 2.3. “Cardio-Ankle Vascular Index” (CAVI) Measurement

CAVI measurements of all study participants were performed using the Fukuda Denshi VS-1500N (Fukuda Denshi Co., Ltd., Tokyo, Japan) device after confirming proper device calibration prior to measurement. Initially derived from the hardness parameter (β) introduced by Hayashi et al., CAVI is computed as follows: “CAVI = a[(2ρ/ΔP) × ln(Ps/Pd) × PWV^2^] + b”. The variables in the provided equation are Ps for systolic blood pressure, Pd for diastolic blood pressure, PWV for pulse wave velocity (“from the root of the aorta to the ankle”), ΔP for pulse pressure (“Ps-Pd”), ρ for blood viscosity, and constants a and b [14]. Simultaneous blood pressure, electrocardiography and cardiac phonographic monitoring were performed on a person who was placed in the supine position. Afterwards, the necessary components for the calculation of CAVI were obtained. Using sphygmomanometer cuffs affixed to bilateral upper arms and ankles, systolic and diastolic blood pressures were measured.

### 2.4. Transthoracic Echocardiographic Measurements

In the study, echocardiographic measurements were made with the General Electronics Medical Systems Echocardiography Vivid 7© device (GE Vingmed Ultrasound AS, Horten, Norway). Pulse wave Doppler (PW) was used to evaluate the speed of blood flow through the mitral valve. It was defined as the velocity e wave (cm/s) obtained during the LV passive filling phase. The LV wall motion was observed through “tissue Doppler” (TDI), with the septal e’ wave (cm/s) extracted from the tissue Doppler wave at the septal part of the mitral annulus. Correspondingly, the lateral e’ wave (cm/s) was obtained from the tissue Doppler wave at the lateral part of the mitral annulus. The evaluation of LV wall relaxation movements secondary to the flow from the mitral valve during diastole was calculated with the formula E/e’. Consistent with current guideline recommendations, tissue Doppler velocities <8 cm/s and E/e′ values >14 were used as predefined thresholds for impaired diastolic function. Furthermore, E/e′ was additionally analyzed as a continuous variable.

### 2.5. Statistical Analysis

The SPSS 25.0 software package (“IBM SPSS Statistics 25, Armonk, NY, USA: IBM Corp. “) was employed for the analysis of the data. Continuous variables are articulated as mean ± standard deviation, and the distribution is described by median (“min–max values”). Categorical variables are expressed as numbers and percentages. Assessment of the data’s normal distribution conformity involved the use of Shapiro–Wilk and Kolmogorov–Smirnov tests. If the assumptions of parametric tests were satisfied, the significance test for the difference between two means was utilized for comparing independent group differences; however, when these assumptions were not met, the Mann–Whitney U test was employed. The chi-square test was utilized to analyze differences between categorical variables. The relationships between numerical variables were investigated using Spearman correlation analysis. Multiple linear regression analysis was used to examine the variables thought to have an impact on CAVI values. In all analyses, a significance level of *p* < 0.05 was used to determine statistical significance.

## 3. Results

Both groups were similar regarding age, gender, height, weight, BMI, MABP and smoking exposure. When the groups were compared, the kidney transplant (KT) group was more hypertensive and hyperlipidemic than the control group. In the KT group, the mean GFR was 62.52 ± 22.23, whereas the control group had a mean GFR of 100.43 ± 16.73 (Table 1).

Although the mean CAVI values were higher in the KT group than in the control group, no statistically significant difference was detected (Table 2). The KT group displayed a significantly lower FGF-23 level compared to the control group. No significant difference was detected between the groups at the klotho level (Table 2). Compared to the control group, the KT group demonstrated significantly higher values in the following: “LV mass index, LV End Systolic Volume Index, LA volume, LAVI, and E/e’“. There is no statistically significant difference between the two groups regarding arterial strain values, but it was lower in the KT group than in the control group (Table 2).

When examined on the basis of the whole cohort, it was observed that CAVI values showed a significant positive correlation with the following: “age, smoking exposure time, SBP, DBP, MABP, pulse pressure, CRP, LV mass index, LV End Systolic Volume Index and E/e’“. A negative correlation was observed with klotho, close to the limit of statistical significance (Table 3).

When the CAVI value of 8.0 m/s and above was considered pathological and the groups were evaluated, it was determined that a total of 34 people, 10 in the control group and 24 in the transplant group, had pathological CAVI values (Table 4).

The KT group was examined according to high (≥8) and low (<8) CAVI levels. The following values were significantly higher in the high-CAVI group: “Age, SBP, pulse pressure, LV mass index, LA volume, LAVI and E/e’“. Arterial strain values were significantly lower in the high-CAVI group than in the low-CAVI group (Table 5).

In multivariable linear regression analysis including age, CKD-EPI, E/e’, klotho, MABP, smoking exposure, CRP and LV mass index, higher E/e’ and lower klotho levels were independently associated with CAVI in the transplant group (Table 6).

## 4. Discussion

Due to limited knowledge in the literature, we examined the association between the level of arterial stiffness determined by CAVI measurement, inflammatory parameters that may be responsible for atherosclerotic mechanisms, and echocardiographically assessed LV function and structure in renal transplant recipients and non-CKD control subjects without clinically overt heart disease. Renal transplantation remains within the CKD spectrum. Cardiovascular risk is high in those patients due to the increased atherosclerotic process and deleterious effects of the immunosuppressive drugs used. Despite advances in cardiovascular risk management, cardiovascular disease remains a threat to graft and patient survival after transplantation. Traditional management of cardiovascular risk factors remains the cornerstone of prevention, but the addition of transplant-specific risk calculators and cardiovascular biomarkers to minimize cardiovascular damage can go some way to overcoming this [15].

CAVI can be easily measured in clinical settings and shows high reproducibility, which may reduce measurement variability and increase its reliability. In particular, PWV neglects the ascending aorta, which is the most distensible aortic segment and plays an important role in ventricular–arterial interaction, whereas CAVI uses the heart-to-ankle transit time, which includes both the aorta (from the heart to aortic bifurcation) and a long muscular artery segment (femoral to ankle). Because of these differences, data on PWV cannot be easily extrapolated to CAVI, and further studies focusing on the role of CAVI as a prognostic biomarker are needed [16]. In the study by Covic et al., arterial stiffness was measured in 41 hemodialysis patients; renal transplantation was performed in 20 patients in the follow-up and it was observed that arterial stiffness decreased in this group as of the third month, while no improvement was observed in the group continuing dialysis [17]. As per the manufacturer’s guidelines, normalcy was defined with a CAVI below 8.0 m/s, and a CAVI of 8.0 m/s or higher was interpreted as a potential indication of suspected arteriosclerosis [18]. When the CAVI value of 8.0 m/s and above was considered pathological and the groups were evaluated, it was determined that a total of 34 people, 10 in the control group and 24 in the transplant group, had pathological CAVI values (Table 4). That is, 70.60% of people with pathologic CAVI were in the transplant group and 29.4% in the control group (*p* = 0.077). Although a higher proportion of pathological CAVI values was observed in the transplant group, this difference did not reach statistical significance. This suggests that some improvements in endothelial dysfunction occur with the improvement of renal function after renal transplantation. Also, smoking cessation after renal transplantation; eliminating the uremic environment; and changing lifestyle, especially providing patients with a more optimal level in terms of nutrition, appear to be important factors that may promote improvement in CAVI values.

In the overall cohort, the significant positive correlations between CAVI and age, blood pressure, and smoking exposure duration support the well-established association of arterial stiffness with cumulative vascular risk burden. Moreover, in the KT subgroup, patients with high CAVI values (>8 m/s) exhibited significantly higher age, systolic blood pressure, and pulse pressure compared with those with low CAVI values (<8 m/s), highlighting the potential contribution of vascular aging and adverse hemodynamic factors to increased arterial stiffness after transplantation. High body mass index after transplantation contributes to the pathogenesis of transplant hypertension, including primary kidney disease, delayed graft function, donor organ quality, calcineurin inhibitor therapy, acute rejection, transplant renal artery stenosis, glucocorticoids, and chronic allograft nephropathy [7,8,9]. Takaki et al., Okura et al., and Kadota et al. reported that CAVI correlated with blood pressure [19,20,21]. Our study identified a positive correlation between CAVI and blood pressure and emphasizes that hypertension becomes more evident as arterial stiffness develops. Noike et al. [22] reported that smoking elevated CAVI, and intriguingly, CAVI decreased after smoking cessation. Kidney recipients who smoke face an augmented risk of CVD death. In conclusion, aging is an uncontrollable process, yet efforts can be made to minimize potential CVD risks by controlling blood pressure and quitting smoking. Therefore, measuring and monitoring CAVI may be a good indicator to reduce and prevent comorbidities.

Various factors such as hypertension, aging, chronic kidney disease, diabetes mellitus, arterial stiffness, and metabolic syndrome are considered causes of LV diastolic dysfunction [23]. Arterial stiffness, increased vascular inflammation, and atherosclerosis are intertwined mechanisms. With atherosclerosis and coronary ischemia leading to a narrowing of the coronary artery lumen, the relaxation function of the heart is damaged before systolic function, resulting in diastolic dysfunction. Cardiac changes begin early in chronic kidney insufficiency. Starting from the first years, changes begin in both the volume and mass of the LV. Myocardial fibrosis develops with increased LV mass. Diastolic dysfunction is observed as a result of an increase in ventricular mass due to increased pressure and volume load in the left ventricle, and subsequent myocardial fibrosis [24]. In our study, the renal transplant group exhibited significantly higher values in the following: “LV mass index, LV End Systolic Volume Index, LA volume, LAVI, and E/e’“. In addition, it was observed that CAVI values showed a significant positive correlation with “LV mass index, LV End Systolic Volume Index and E/e’“ in the entire cohort. In conclusion, although improvement in systolic function and regression in LVH and dilatation are expected with the correction of uremia, reduction in fluid load and closure of the av fistula with renal transplantation, it still indicates that the risk remains higher than in the healthy population. It shows that the atherosclerotic process is active in the CKD spectrum in these patients and that cardiovascular risks also continue.

The effective treatment of ESRD is renal transplantation, and studies on the impact of improved renal function on cardiovascular mortality, morbidity and increased arterial stiffness in these patients are ongoing. In this study, results showed a positive correlation of CAVI with E/e’ in both the entire cohort and the KT group. Increased E/e’ and low klotho were the parameters determining CAVI in the linear regression analysis. These data suggest a strong correlation between the blood pressure-independent arterial stiffness parameter CAVI and LV diastolic dysfunction. Based on our results, patients with LV diastolic dysfunction may require further evaluation and therapeutic intervention for arterial stiffness.

No study identified a significant correlation between FGF-23 levels and CAVI measurements. The lack of association between FGF-23 and arterial calcification suggests that FGF-23 does not contribute to the promotion of arterial calcification. However, in our study, serum klotho and CAVI values showed a negative correlation close to the limit of statistical significance in the whole cohort (Table 3). Additionally, multivariable analysis demonstrated that higher E/e’ and lower klotho levels were independently associated with increased CAVI in the transplant group (Table 6). Although the correlation between klotho and CAVI in the whole cohort did not reach statistical significance, the independent association observed in multivariate analysis within the transplant group suggests that this relationship may be influenced by confounding variables. The association between higher E/e’ and arterial stiffness likely reflects the interplay between diastolic dysfunction and vascular compliance, suggesting a shared pathophysiological pathway involving increased left ventricular filling pressures and vascular remodeling. In parallel, the inverse relationship between klotho levels and CAVI supports the emerging role of klotho as a vasculoprotective factor, potentially mediated through its anti-inflammatory, anti-oxidative, and endothelial-preserving effects. These findings highlight the combined contribution of cardiac diastolic burden and impaired mineral metabolism-related pathways to arterial stiffness in kidney transplant recipients. Our results support that the correlation between low serum klotho levels and vascular dysfunction, particularly arterial stiffness, is evident, although few studies have investigated its role in the field of renal transplantation. Further research is needed to shed light on the complex mechanisms of klotho in KT patients for diagnosis and treatment purposes.

Additionally, Provenzano et al. highlighted that the renal resistive index (RI), a Doppler-derived parameter reflecting intrarenal vascular resistance, is closely associated with cardiovascular comorbidities, impaired renal function, and systemic vascular damage in patients with chronic kidney disease. In this context, RI is increasingly recognized not merely as a marker of renal hemodynamics but as an integrative indicator of systemic vascular dysfunction. The observed associations between RI and factors such as diabetes, cardiovascular disease, and serum phosphorus further support the concept that renal microvascular alterations are strongly influenced by systemic atherosclerotic and metabolic processes [25]. In line with these findings, our results demonstrating a relationship between increased arterial stiffness (CAVI), elevated filling pressures (E/e’), and reduced klotho levels may reflect a shared pathophysiological pathway characterized by endothelial dysfunction, vascular remodeling, and disturbed mineral metabolism. Taken together, these data reinforce the notion that cardiovascular and renal dysfunction in this population are tightly interconnected, and that vascular biomarkers—whether derived from Doppler indices such as RI or stiffness measures such as CAVI—capture overlapping aspects of this complex interaction.

This study has several limitations. First, the inclusion of diabetic patients in the transplant group reflects real-world clinical practice; however, diabetes may act as a confounding factor in the evaluation of arterial stiffness and cardiac function. Second, the absence of pre-transplant CAVI and klotho measurements precludes a definitive assessment of temporal changes in arterial stiffness and klotho dynamics following transplantation, thereby limiting causal inferences regarding the progression or regression of the atherosclerotic process in the stable post-transplant period. Furthermore, the cross-sectional nature of the analysis restricts our ability to establish directionality between the observed associations. Future prospective, longitudinal studies incorporating serial assessments of vascular and biochemical parameters are warranted to better elucidate these relationships and to clarify their potential implications for clinical risk stratification, as well as the development of targeted preventive and therapeutic strategies.

## 5. Conclusions

Our results reveal that high CAVI scores, i.e., arterial stiffness, persist even after successful renal transplantation, albeit with some reduction, and that low klotho and the impaired diastolic function parameter E/e’ are independently associated with CAVI. In this context, CAVI may serve as a complementary tool for assessing the burden of arterial stiffness in kidney transplant recipients. Our study also highlights the relationship between echocardiographically assessed diastolic dysfunction and arterial stiffness measured by CAVI. We further demonstrated that CAVI levels were significantly associated with LVMI, suggesting that cardiac hypertrophy may be partially related to the observed ventricular–arterial interaction. Taken together, these findings indicate the persistence of cardiovascular dysfunction after kidney transplantation and underscore the importance of strict cardiovascular risk control in this population. They also suggest that klotho-related pathways and arterial stiffness may represent clinically relevant targets for further investigation in the context of early cardiovascular risk assessment and prevention of cardiovascular complications.

## Figures and Tables

**Table 1:** Demographic characteristics and laboratory findings of the groups.

	Control Group (*n* = 55)	Transplant Group (*n* = 75)	*p* Value
**Age (years)**	45.25 ± 13.27	48.01 ± 12.12	0.22
**Gender (w/m)**	30/25	36/39	0.461
**BMI (kg/m^2^)**	29.06 ± 5.22	27.84 ± 4.97	0.177
**Transplant time (month)**	-	103.21 + 54.82	-
**Smoking exposure (package/year)**	14.96 ± 13.25	15.05 ± 12.43	0.945
**HT (y/n)**	12/43	53/22	**0.0001**
**HL (y/n)**	2/53	23/52	**0.0001**
**DM (y/n)**	-	21/54	-
**MABP (mmHg)**	96.05 ± 8.77	99.95 ± 10.89	0.071
**GFR-CKD-EPI (mL/min/1.73m^2^)**	100.43 ± 16.73	62.52 ± 22.23	**0.0001**
**SERUM CRP (mg/dL)**	0.33 ± 0.41	0.52 ± 0.69	**0.053**
**LDL CHOLESTEROL (mg/dL)**	107.58 ± 33.95	100.35 ± 30.32	0.204
**SERUM CREATININE (mg/dL)**	0.79 ± 0.16	1.34 ± 0.55	**0.0001**
**SERUM URIC ACID (mg/dL)**	4.79 ± 1.02	5.67 ± 1.06	**0.0001**
**SERUM CALCIUM (mg/dL)**	9.56 ± 0.4	9.44 ± 0.63	0.18
**SERUM PHOSPHORUS (mg/dL)**	3.75 ± 0.39	3.47 ± 0.67	**0.002**
**SERUM ALBUMIN (g/L)**	4.53 ± 0.38	4.37 ± 0.4	0.093
**SERUM PTH (pg/m** **L** **)**	51.91 ± 13.68	101.79 ± 73.8	**0.0001**
**PROTEINURIA (mg/day)**	213.63 ± 158.41	610.55 ± 886.04	**0.021**
**25-HYDROXY VIT D (ug/L)**	21.02 ± 11.99	21.38 ± 9.92	0.52

HT, hypertension; DM, diabetes mellitus; HL, hyperlipidemia; BMI, body mass index; MABP, mean arterial blood pressure; *p* < 0.05 is statistically significant.

**Table 2:** CAVI and atherosclerotic markers, and comparison of echocardiographic parameters of groups.

	Control Group (*n* = 55)	Transplant Group (*n* = 75)	*p* Value
**CAVI**	7.02 ± 1.3	7.26 ± 1.67	0.81
**FGF-23** **(pg/mL)**	75.3 ± 134.92	71.14 ± 125.16	**0.018**
**s-Klotho(ng/mL)**	3.71 ± 6.84	4.18 ± 6.84	0.246
**LVEF %**	61.3 ± 5.87	61.21 ± 6.34	0.651
**LV MASS INDEX (g/m^2^)**	85.05 ± 19.34	103.89 ± 23.92	**0.000**
**LV END SYSTOLIC VOLUME INDEX** **(ml/m^2^)**	19.48 ± 4.75	23.9 ± 6.09	**0.0001**
**ARTERIAL STRAIN**	7.41 ± 3.62	6.33 ± 3.78	**0.069**
**LAVI**	22.25 ± 6.85	28.7 ± 9.5	**0.000**
**E/e’**	6.93 ± 1.79	8.65 ± 2.88	**0.001**

CAVI, cardio-ankle vascular index; FGF-23, C-terminal fibroblast growth factor-23; s-Klotho, soluble alpha klotho; LVEF, left ventricular ejection fraction; LV, left ventricular mass; LA, left atrial; LAVI, left atrial volume index; E/e’, e-deceleration time; *p* < 0.05 is statistically significant.

**Table 3:** Correlation of demographic characteristics and laboratory parameters with CAVI in whole cohort.

CAVI (*n* = 130)	R Value	*p*-Value
**Age (years)**	0.465	**0** **.** **000**
**Smoking exposure (package/year)**	0.317	**0.018**
**MABP (mmhg)**	0.255	**0.003**
**CKD.EPI (ml/dL/1.73 m^2^)**	−0.06	0.528
**Serum calcium (mg/dL)**	0.126	0.153
**Serum PTH (pg/mL)**	0.078	0.378
**LV MASS INDEX (g/m^2^)**	0.476	**0** **.** **000**
**LV END SYSTOLIC** **VOLUME INDEX**	0.254	**0.011**
**E/e’**	0.505	**0** **.** **000**
**s-Klotho (ng/mL)**	−0.166	**0.059**

*p* < 0.05 is statistically significant.

**Table 4:** CAVI distribution by group.

CAVI		Control (*n* = 55)	Transplant (*n* = 75)	Total	Intergroup *p*
<8	Number	45	51	96	0.077
	Percentage value	46.90%	53%	100.00%	
	Intergroup percentage value	81.80%	68.00%	73.80%	
≥8	Number	10	24	34	
	Percentage value	29.40%	70.60%	100.00%	
	Intergroup percentage value	18.20%	32.00%	26.20%	
Total		100.00%	100.00%	130	

*p* < 0.05 is statistically significant.

**Table 5:** Comparison of demographic, laboratory and atherosclerotic parameters of patients in the transplant Group by their CAVI Scores.

	Below 8 (*n* = 51)		8 and Above (*n* = 24)		*p* Value
**Age (years)**	43.47 ± 10.8	46 (21–71)	57.67 ± 8.7	58 (39–74)	**0.0001**
**Bmi (kg/m^2^)**	28.22 ± 5.38	27.61 (19.07–44.54)	27.03 ± 3.93	27.55 (20.82–38.54)	0.46
**CAVI**	6.33 ± 0.95	6.5 (3.15–7.9)	9.24 ± 1.01	8.9 (8.25–12.8)	**0.0001**
**MABP (mmhg)**	98.49 ± 10.26	96.67 (80–131.17)	103.05 ± 11.75	102.58 (81.83–124.67)	0.34
**CKD.EPI (ml/dL/1.73 m^2^)**	61.05 ± 21.8	61.5 (20–121)	65.45 ± 23.36	67 (23–104)	0.48
**Serum PTH (pg/mL)**	106.1 ± 78.37	75.45 (25–398)	92.82 ± 63.87	73.22 (9.8–253)	0.56
**s-Klotho (ng/mL)**	4.34 ± 6.97	0.33 (0.1–20)	3.83 ± 6.67	0.41 (0.1–20)	0.78
**LV MASS INDEX (g/m^2^)**	96.64 ± 21.33	91.87 (51.87–141.76)	119.3 ± 22.1	124.55 (74.2–161.95)	**0.0001**
**ARTERIAL STRAIN**	7.41 ± 5.37	6.49 (1.48–34.02)	7.15 ± 10.74	3.73 (0.92–53.57)	**0.048**
**LAVI**	25.78 ± 8.6	25.39 (12.75–64.99)	34.91 ± 8.41	33.42 (15.01–55.83)	**0.0001**
**E/e’**	7.64 ± 2.41	7.24 (2.77–15.46)	10.75 ± 2.69	9.77 (7.21–18.33)	**0.0001**

*p* < 0.05 is statistically significant.

**Table 6:** Linear regression analysis results by CAVI in the transplant and control groups.

Group		Standardized Beta	t Value	*p* Value	Confidence Intervals	
					Lower Limit	Upper Limit
**Control**	**Age (years)**	−0.014	−0.041	0.968	−0.071	0.068
	**GFR-CKD-EPI (ml/dk/1.73 m^2^)**	0.258	0.906	0.381	−0.035	0.086
	**E/e’**	0.427	1.287	0.22	−0.171	0.676
	**s-Klotho (ng/mL)**	−0.242	−0.763	0.459	−0.259	0.124
	**MABP (mmhg)**	−0.28	−1.256	0.231	−0.115	0.03
	**Smoking exposure** **(package/year)**	0.122	0.535	0.601	−0.033	0.055
	**CRP (mg/dL)**	0.199	0.664	0.519	−1.499	2.829
	**LV MASS INDEX (g/m^2^)**	0.331	1.172	0.262	−0.017	0.058
**Transplant**	**Age (years)**	0.154	1.006	0.327	−0.02	0.056
	**GFR-CKD-EPI (ml/dk/1.73 m^2^)**	0.13	1.19	0.249	−0.007	0.025
	**E/e’**	0.587	2.781	**0.012**	0.063	0.447
	**s-Klotho (ng/mL)**	−0.544	−4.132	**0.001**	−0.241	−0.079
	**MABP (mmhg)**	0.021	0.163	0.872	−0.037	0.043
	**Smoking exposure** **(package/year)**	−0.147	−0.957	0.35	−0.059	0.022
	**CRP (mg/dL)**	−0.058	−0.441	0.664	−0.619	0.404
	**LV MASS INDEX (g/m^2^)**	0.3	1.556	0.136	−0.008	0.054

Control group R square: 0.799; transplant group R square: 0.502; *p* < 0.05 is statistically significant.

## Data Availability

The data in this study are available from the corresponding author upon reasonable request.
